# The Dual Luminescence Lifetime pH/Oxygen Sensor: Evaluation of Applicability for Intravital Analysis of 2D- and 3D-Cultivated Human Endometrial Mesenchymal Stromal Cells

**DOI:** 10.3390/ijms242115606

**Published:** 2023-10-26

**Authors:** Ilia K. Litvinov, Tatiana N. Belyaeva, Anna V. Salova, Nikolay D. Aksenov, Pavel S. Chelushkin, Anastasia I. Solomatina, Sergey P. Tunik, Elena S. Kornilova

**Affiliations:** 1 Institute of Cytology, Russian Academy of Sciences, Tikhoretsky av. 4, 194064 Saint-Petersburg, Russia; lik314@mail.ru (I.K.L.); tatbelyaeva@gmail.com (T.N.B.); avsalova@gmail.com (A.V.S.); aksenovn@gmail.com (N.D.A.); 2 Institute of Chemistry, St. Petersburg State University, Universitetskii av., 26, 198504 Saint-Petersburg, Russia; p.chelushkin@spbu.ru (P.S.C.); nastisol@gmail.com (A.I.S.); 3 Higher School of Biomedical Systems and Technologies, Peter the Great St. Petersburg Polytechnic University, Khlopina Str. 11, 195251 Saint-Petersburg, Russia

**Keywords:** dual sensor, FITC, Ir(III) complexes, fluorescence, phosphorescence, lifetime imaging, autofluorescence, oxygen sensing, hypoxia, pH sensing, endolysosomes, mesenchymal stromal cells, spheroids

## Abstract

The oxygenation of cells and tissues and acidification of the cellular endolysosomal system are among the major factors that ensure normal functioning of an organism and are violated in various pathologies. Recording of these parameters and their changes under various conditions is an important task for both basic research and clinical applications. In the present work, we utilized internalizable dual pH/O_2_ lifetime sensor (Ir-HSA-FITC) based on the covalent conjugation of human serum albumin (HSA) with fluorescein isothiocyanate (FITC) as pH sensor and an orthometalated iridium complex as O_2_ sensor. The probe was tested for simultaneous detection of acidification level and oxygen concentration in endolysosomes of endometrial mesenchymal stem/stromal cells (enMSCs) cultivated as 2D monolayers and 3D spheroids. Using a combined FLIM/PLIM approach, we found that due to high autofluorescence of enMSCs FITC lifetime signal in control cells was insufficient to estimate pH changes. However, using flow cytometry and confocal microscopy, we managed to detect the FITC signal response to inhibition of endolysosomal acidification by Bafilomycin A1. The iridium chromophore phosphorescence was detected reliably by all methods used. It was demonstrated that the sensor, accumulated in endolysosomes for 24 h, disappeared from proliferating 2D enMSCs by 72 h, but can still be recorded in non-proliferating spheroids. PLIM showed high sensitivity and responsiveness of iridium chromophore phosphorescence to experimental hypoxia both in 2D and 3D cultures. In spheroids, the phosphorescence signal was detected at a depth of up to 60 μm using PLIM and showed a gradient in the intracellular O_2_ level towards their center.

## 1. Introduction

Multipotent mesenchymal stem/stromal cells (MSCs) are found in almost all connective tissues of adult organisms [1]. Their ability for self-renewal and differentiation, as well as the secretion of cytokines and signaling hormones in particular, with angiogenic and anti-apoptotic effects, makes them promising tools for biomedical applications [2].

The use of monolayer cultures, including MSCs, has become widespread as a model that makes it easy to manipulate the state of these cells by adding various agents to the culture medium and studying the evoked cellular responses. A monolayer can be considered a two-dimensional (or 2D) system in which each cell has the same access to nutrients and signaling molecules, which ensures uniform behavior of the entire cell population during the experiment. However, the cells in tissues, depending on their position in the three-dimensional (3D) structure characterized by natural gradients of oxygen, signaling molecules, nutrients, and metabolic products, have unequal access to these vital substances, which affects the intracellular processes in individual cells. Obviously, a 3D configuration is much more physiological, and 3D culture systems are actively introduced into experimental practice in order to mimic natural conditions. It should be emphasized that studies of this kind are mainly devoted to spheroids obtained from tumor cells, while the behavior of MSC-derived spheroids is analyzed to a significantly lesser extent. Suffice it to say that more than 21 thousand publications on tumor spheroids and less than 400 on those obtained from MSCs are cited in PubMed up to now. However, recent studies show (see [3] for a review) that 3D MSCs demonstrate increased stemness, regenerative potential, and other benefits compared to those cultured in 2D. Thus, understanding these differences is extremely important for both fundamental research and practical applications such as regenerative and reparative medicine.

One of the key factors affecting cellular metabolism is the level of available oxygen, since O_2_ is a basic substrate for mitochondrial ATP production and numerous intracellular redox reactions also taking place both in the cytosol and in membrane compartments such as lysosomes. It is important to note that the metabolic environment of the niche in which MSCs usually grow is characterized by low oxygen tension [4], therefore cells cultured under such conditions exhibited increased proliferation, migration, and angiogenesis, and decreased senescence and apoptosis [5,6,7]. It has been shown [8] that cell populations derived from MSCs demonstrate higher proliferative activity when cultivated at low oxygen tension (2 to 5% O_2_, corresponding to 15 to 38 mmHg), compared to normal oxygen tension (21% O_2_, corresponding to 160 mmHg, according to [9]). In general, the cells in tissue layers more distant from vessels normally exist under conditions of moderate, or physiological, hypoxia. There are also reports on inherent hypoxia in some tumors [10], and distinguishing of physiological levels of hypoxia from those typical for pathologies can be of great value in identifying certain diseases.

Another very significant factor in the normal activity of cells of all types is the level of acidification of the endolysosomal apparatus, provided by the action of the vesicular proton pump V_0_/V_1_ [11,12]. It is important to note that MSC senescence and various pathologies are characterized by changes in both endolysosome acidification [13,14] and ROS production in lysosomes [15]; however, understanding of links between these two factors is still obscure. Obviously, knowledge of the actual levels and local microgradients of O_2_ and pH at the cellular level and their precise control in vitro and in vivo in complex 2D and 3D cell models is currently an urgent task in biology and medicine. Consequently, appropriate tools are needed to solve it, and the development and characterization of various biosensors is a hot spot of modern biomedical research.

In this context, the sensors allowing simultaneous determination of several parameters, for example, pH level and oxygen pressure, in the same intracellular structures are of particular interest. Phosphorescent cyclometallated Ir(III) complexes are a convenient basis for creating highly sensitive biolabels and sensors that visualize the O_2_ level in tissues [16,17,18]. It is also worth noting that cyclometallated Ir(III) complexes usually remain stable under exposure to photoexcitation that makes them suitable candidates for prolonged biological experiments. The probe response in the imaging and sensing measurements with the phosphorescent Ir(III) complexes can be quantified in two modes, namely either by using a ratiometric approach [19] or by phosphorescence lifetime imaging (PLIM) [20]. However, the latter has some evident advantages due to the independence of the probe signal from its concentration and optical properties of the sample under study, thus giving high-resolution images at cellular level and providing accurate information about the microenvironment along with real-time changes in O_2_ levels. As for the estimation of media acidity, numerous fluorescent organic probes are commercially available, among which fluorescein is widely used because its intensity decreases with increasing acidity of the microenvironment [21].

Despite the fact that dual pH/O_2_ sensors based on ratiometric sensing mode are well-known [22,23,24], the development of their lifetime-based analogs, which are more promising due to higher measurements reliability, appeared to be more challenging [25]. Recently, we have developed the dual luminescence pH/O_2_ lifetime sensor (Ir-HSA-FITC) based on the covalent conjugation of fluorescein in the form of fluorescein isothiocyanate (FITC, fluorescent pH sensor) and an orthometalated iridium complex (phosphorescent O_2_ sensor) to human serum albumin (HSA) [26]. The emission and excitation spectra of the dual sensor represent a linear combination of the corresponding spectroscopic patterns of individual chromophores (fluorescein and iridium complex), while overlapping excitation spectra allow pumping of both emitters at 405 nm. The location of the sensor emission components (fluorescein fluorescence centered at 520 nm and structured phosphorescence of Ir band at 580–720 nm) provides independent acquisition of the fluorescence and phosphorescence decays in the corresponding spectral windows. Moreover, phosphorescence signal is also separated from that of fluorescence in “time domain” mode of lifetime measurements that additionally increase sensor selectivity to the target parameters. The dual sensor features mentioned above make possible application of the advanced lifetime microscopy technique, namely the combined FLIM/PLIM mode of the data acquisition, which allows simultaneous spatially co-localized recording of the fluorescence and phosphorescence lifetimes in one pulse sequence [26].

Linking these two chromophores to HSA increases the overall size of the sensor to the value of ca. 8 nm (i.e., to the size of HSA) that, in turn, makes endocytosis the only route for its entry into the cells [27,28]. Theoretically, in this case, the dual sensor will be localized preferentially in endosomes and lysosomes that allow avoiding a high fluorescence background of cytoplasmically located sensor, and makes possible detection of pH and O_2_ levels and their external condition-generated changes in the same endolysosomal structures. In the case of intracellular measurements in combined FLIM/PLIM mode, the independence of the fluorophore and phosphor lifetime from the probe concentration becomes quite beneficial, since the number of sensor molecules can vary greatly in individual endosomes and lysosomes and can differ at different stages of endocytosis.

However, practical implementation of any sensor needs detailed analysis of possible limitations of its application and optimization of experimental protocols for both cultured cell models and tissues of living organisms to avoid misinterpretations of obtained data. The supposed limitations of the dual sensor may be related to two main points. First, the cells possess various endogenous physiologically relevant molecules (such as FAD [29], or NAD(P)H [30]) responsible for so called autofluorescence that can also be recorded by FLIM technique, and its level can compromise measurements of exogenously added fluorophores. Second, endolysosomal system organization is dependent of the cell type which can be different in the number, size and localization of endolysosomes, as well as concentration of fluorophore inside them.

In this article, we have analyzed in detail the possibility of application of the internalizable Ir-HSA-FITC dual sensor for the simultaneous detection of the acidification level and oxygen concentration in human endometrial MSC (enMSC) endolysosomes in a monolayer and in spheroids as the models of 2D- and 3D-cell growth. Endolysosomes in enMSCs are smaller and their number is higher than in the cells like CHO and A549 and are characterized by higher level of autofluorescence in green region of spectrum. Using monolayer enMSCs and enMSC spheroids models, we have performed the experiments aimed at elucidating cellular uptake, intracellular distribution, and excretion of Ir-HSA-FITC probe and possibility to monitor the level of pH and O_2_ consumption by using several approaches such as confocal microscopy, flow cytometry, and a combined FLIM/PLIM technique. We have found that high autofluorescence level compromise FLIM data, but accumulation of FITC component is perfectly recorded by cytofluorometry. We believe that autofluorescense level must be taken into account as important parameter that can influence interpretation of acquired data. At the same time, PLIM experiments make possible monitoring of fast variations in O_2_ level both in monolayer cells and in spheroids at a spheroid depth greater than that allowed by confocal microscopy. Our findings are critical to understanding what dual sensor design improvements are needed to achieve a more versatile product.

## 2. Results

### 2.1. Ir-HSA-FITC Sensor in Monolayer enMSCs: Spectral and Luminescent Characteristics and Intracellular Localization

**Figure 1:**
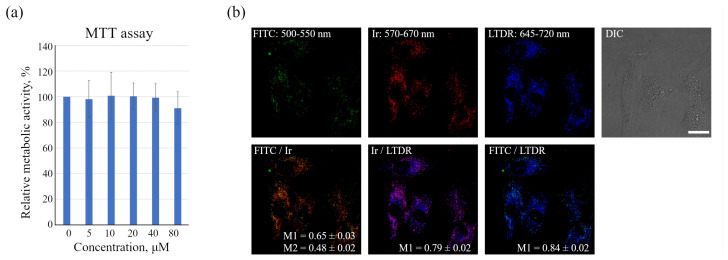
Ir-HSA-FITC interaction with monolayer enMSCs. (**a**) MTT analysis of the metabolic activity of enMSCs after 24 h of incubation with Ir-HSA-FITC. The optical density of formazan produced by the metabolic active control cells not incubated with Ir-HSA-FITC was taken to be 100%. The results are presented as a mean ± SEM. (**b**) Intracellular distribution of Ir-HSA-FITC (20 μm, 24 h) in enMSCs (FITC—green, Ir—red) stained with LysoTracker Deep Red (LTDR, 50 nM, 15 min, blue). Manders’ co-localization coefficients (M) are presented as a mean ± SEM. DIC—differential interference contrast images. Scale bar 20 μm.

**Figure 2:**
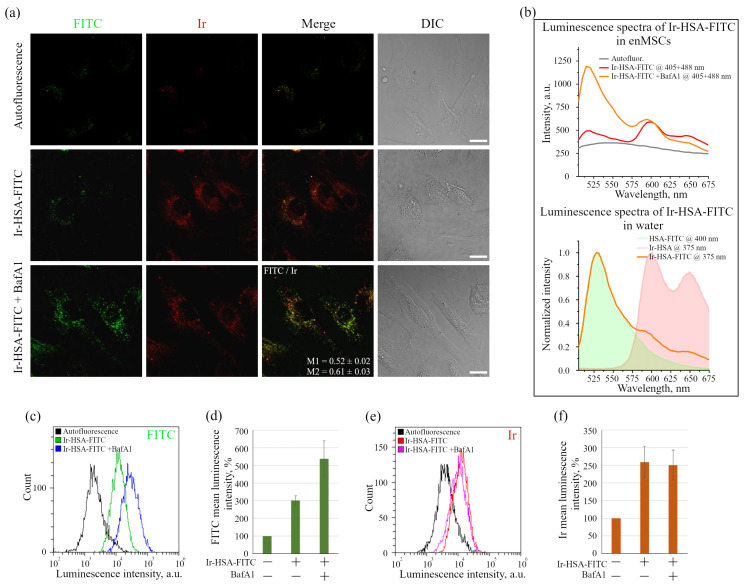
Luminescence of Ir-HSA-FITC (20 μm, 24 h) in enMSCs before and after addition of BafA1 (100 nM, 30 min). (**a**) Confocal images of enMSCs in green (FITC; 500–550 nm) and red (Ir; 570–670 nm) channels of luminescence and differential interference contrast (DIC). Control cells—autofluorescence. The images represent a projection of Z-stack onto a single image obtained by max intensity method (ImageJ). Representative images of the cells are presented. Scale bars 20 μm. (**b**) Luminescence spectra of Ir-HSA-FITC in enMSCs were obtained using spectral scanning mode (Mode/xyλ) of cells in the range of 505–675 nm with a step of 5 nm (luminescence excitation was carried out simultaneously by two lasers at 405 and 488 nm). Spectra were taken from the selected areas of the same size enriched in vesicles for every case (upper graph). For comparison, the spectra of HSA-FITC, Ir-HSA and Ir-HSA-FITC in water are presented in the lower graph (laser excitation wavelengths are indicated in the legend). (**c**–**f**) Flow cytometry analysis of cells after incubation with Ir-HSA-FITC (20 μm, 24 h) without/with BafA1 (100 nM, 30 min). Distributions of the luminescence intensity of cell populations in the FITC (**c**) and Ir (**e**) channels and the histograms of FITC (**d**) and Ir (**f**) mean luminescence intensity per cell are presented. The value of the cell luminescence without Ir-HSA-FITC indicates their autofluorescence and is taken as 100%. The results are presented as a mean ± SEM. Three independent experiments were carried out.

The synthesis and photophysical characteristics of the dual Ir-HSA-FITC sensor have been described previously, including the synthetic conditions chosen to obtain the samples with the ratio of Ir:FITC:HSA of approximately 1:1:1 [26]. In agreement with the cytotoxicity data reported previously for the CHO-K1 cells, the MTT test for the enMSC monolayer culture showed that the sensor concentrations up to 80 μm were non-toxic for cells (Figure 1a), and the concentration of 20 μm was enough for FITC and Ir signals detection using confocal microscopy (Figure 1b). Monolayer enMSCs were incubated in the presence of the sensor for 24 h and then luminescence was recorded in live cells in green (FITC) and red (Ir) channels in the ranges indicated in Figure 1b. The obtained spectroscopic pattern fits completely to that one recorded earlier [26] in water (Figure 2b), which is indicative of the sensor remaining intact in the system under study. The emission of both sensor components was detected in vesicular structures with a high degree of co-localization with the lysosome marker LysoTracker Deep Red (LTDR, Figure 1b), which is also consistent with previously obtained data on CHO-K1 cells [26].

It was found that the number of visible FITC-positive vesicles in enMSCs was much less than that of Ir-containing ones, and co-localization of sensors with each other was much lower than with LTDR (Figure 1b). This situation may be the result of a lower brightness of FITC compared to Ir emitter, since the detected FITC is mainly localized in acidified lysosomes after 24 h incubation and it is reasonable to assume that the main part of the FITC chromophores was significantly quenched by the acidic (pH 4.5–5.5) intralysosomal environment, but can still be detected in some vesicles with more basic pH and/or in those where it was most concentrated. Indeed, upon treatment of sensor-containing enMSCs with the proton vacuolar pump inhibitor bafilomycin A1 (BafA1), which inhibits endolysosomal acidification, the brightness and number of FITC-containing vesicles sharply increased (Figure 2a,b). As a result, M1 decreased from 0.65 to 0.52 due to an increase in the number of visible FITC-positive vesicles, and M2 grew from 0.48 to 0.61 due to increased number of endolysosomes with both Ir and FITC signals (Figure 2a). Thus, the signal in the green channel is mainly pH dependent, which made possible to identify it as emission of FITC localized in acidic lysosomes. However, it should be noted that the analysis of intrinsic fluorescence of monolayer enMSCs (upper row of images in Figure 2a) in the absence of the sensor may reveal some faintly (but non-negligibly) luminous vesicular structures in the green, and fewer in the red channels.

This makes it difficult to differentiate vesicles, especially those containing the FITC-like signal in the absence of BafA1, from endogenous chromophores emitting in this region (such as NAD(P)H/NADH, FAD, and lipofuscin) using confocal microscopy of monolayer enMSCs. This phenomenon is associated with some features of enMSCs that distinguish them from the cells such as CHO-K1 or HeLa. Firstly, enMSCs are much more spread and flat, and secondly, their endolysosomal apparatus is represented by a significantly larger number of small endosomes and lysosomes, which are much less concentrated in the perinuclear region that is typical for the above-mentioned cell lines. So, segmentation of images for individual vesicles is highly problematic. In general, these cells are characterized by a very high level of autofluorescence, which cannot be neglected when evaluating the signal. Therefore, the level of autofluorescence was taken into account in all further experiments. Spectral analysis of monolayer enMSCs (Figure 2b) showed that cells incubated with the sensor in the absence of BafA1 demonstrated only a slight excess of the signal over the level of their autofluorescence, especially in the 500–550 nm region, coinciding with the FITC fluorescence peak (515–520 nm), while the signal-to-noise ratio in the Ir channel was substantially higher.

However, when the sensor luminescence was analyzed using the flow cytometry method, which involves rounding the cells after removing them from the substrate, the signal was detected from the whole cell and background autofluorescence was significantly separated from the luminescence of both emitters. The treatment of cells with BafA1 led to an increase in the FITC signal (Figure 2c,d) without affecting the Ir signal intensity (Figure 2e,f). The obtained data suggest that, after 24 h of incubation, the main part of the sensor is concentrated in endolysosomes with the pH range of 4.5–5.5, at which FITC is largely quenched and cannot be practically detected in monolayer enMSCs at the level of individual vesicles, but is reliably detected by flow cytometry analysis as average luminescence intensity of individual cells.

**Figure 3:**
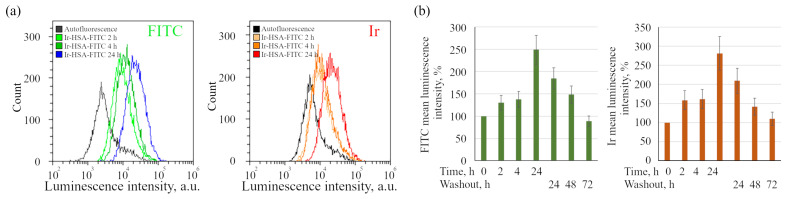
Dynamics of intracellular accumulation of Ir-HSA-FITC (20 μm) by enMSCs obtained by flow cytometry analysis. (**a**) The distributions of luminescence intensity of the enMSC population in FITC and Ir channels before (0 h) and after incubation with Ir-HSA-FITC for 2, 4, 24 h. (**b**) Flow cytometry analysis of Ir-HSA-FITC accumulation (2, 4, 24 h) and washout after media change (24, 48, 72 h) by enMSCs. The histograms of mean luminescence intensity per cell are presented. The value of the cell luminescence without Ir-HSA-FITC indicates their autofluorescence and is taken as 100%. The results are presented as a mean ± SEM. Three independent experiments were carried out.

If the sensor is localized in acidic endosomes after 24 h incubation, then the question arises whether it is possible to acquire the FITC signal in early endosomes, where the pH level is about of 6.8–6.0, by incubating cells with the sensor for a shorter time. Unfortunately, confocal microscopy of monolayer cells did not allow reliable identification of FITC and Ir signals after 2–4 h of incubation with Ir-HSA-FITC (data not shown). It is well known that albumin can bind to membrane proteins of the scavenger family receptors and thus enter the cells by receptor-mediated endocytosis through clathrin-coated pits, but these proteins are expressed mainly by professional phagocytes [28,31]. Thus, the Ir-HSA-FITC sensor enters the cells of other types most likely as a fluid-phase marker by all ways associated with the capture of portions of the external medium. It is known that, contrary to substances entering the cells via receptor-mediated endocytosis, fluid-phase cargoes are poorly concentrated in early endosomes, thus the amount of the sensor in them does not allow reliable detection of early endosomal structures. However, at the level of whole cells, flow cytometry approach reliably detects a slight accumulation of the sensor in enMSCs after 2 and 4 h, although after 24 h it increases in the FITC and Ir luminescence channels by 2.5 and 2.8 times, respectively, relative to the level of autofluorescence (Figure 3).

For long-term experiments, it is essential to understand how long and to what extent the sensor signals remain detectable in cells after its removal from the external medium. In these experiments, the cells after incubation with Ir-HSA-FITC (20 μm, 24 h) were washed to remove the sensor from the external medium and analyzed after 24, 48, and 72 h post washing. The results are presented as a diagram (Figure 3b, washout), obtained on the basis of flow cytometry data as mean luminescence intensity of the sensor per cell. It was found that there is a gradual decrease in fluorescence in the FITC and Ir channels, and by 72 h it drops almost to the control values (Figure 3b). This effect can be explained by dilution of the number of lysosomes during cell division, or elimination of the sensor from the cells by secretion of secondary lysosomes. It is also important to take into account that the data obtained on Ir phosphorescence may reflect not only the amount of sensor in the cells, but also the change in oxygen concentration due to the increase in culture density after 72 h. The latter assumption is unlikely, since the signals change in the same way in both channels, and the variations in the intensity of Ir phosphorescence from normoxia to hypoxia conditions do not exceed ca. 40–60%. However, the used enMSC line is characterized by high proliferation rate with the cell cycle duration to be about 22–24 h, which favors the lysosome dilution hypothesis more. Thus, the changes in the luminescence intensity of the Ir and FITC signals obtained from whole cells in suspension by flow cytometry approach can be used to evaluate the accumulation of the sensor and FITC sensitivity to changes in pH, even in the cells with high autofluorescence levels such as enMSCs.

### 2.2. Characterization of Ir-HSA-FITC Sensor in Monolayer enMSCs by Time-Resolved Microscopy

**Figure 4:**
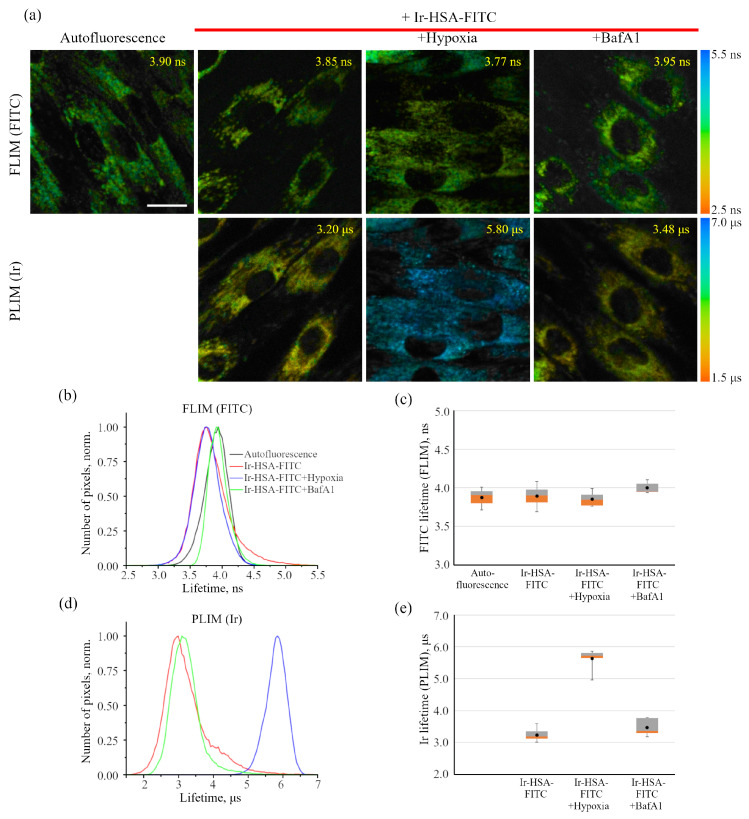
Fluorescence-lifetime (FLIM) and phosphorescence-lifetime (PLIM) imaging microscopy of enMSCs with Ir-HSA-FITC. (**a**) FLIM and PLIM images of enMSCs incubated without (autofluorescence) or with Ir-HSA-FITC (20 μm, 24 h). Ir-HSA-FITC-loaded cells were treated with BafA1 (100 nM, 30 min). Hypoxia was stimulated by substitution of air with nitrogen and the cells were incubated in hypoxic medium for 30 min. The values of the medians of the luminescence lifetimes of Ir-HSA-FITC for each image are presented. Scale bar 20 μm. (**b**,**d**) Distribution histograms of the luminescence lifetime values for Ir-HSA-FITC: FLIM for FITC (**b**), PLIM for Ir (**d**). (**c**,**e**) Box plots of values of the medians of the luminescence lifetimes of FITC fluorescence (**c**) and Ir phosphorescence (**e**) in enMSCs under different conditions from three independent experiments. In box plots, whiskers represent the minimum and maximum values, bases represent the interquartile range between the first (red) and third quartiles (gray), and midlines represent the median. Point symbols indicate the mean value. Three independent experiments were carried out.

As was mentioned above, gradual concentration of the sensor occurs during endocytosis, which also leads to an increase in the luminescence intensity, and may be incorrectly interpreted as a change in the pH or oxygen level in endolysosomes. From this point of view, the luminescence lifetime is a much more reliable parameter, which is independent of the chromophore concentration. Figure 4a shows the combined FLIM/PLIM images of the enMSCs after incubation with Ir-HSA-FITC. Since the number of endolysosomes in enMSCs is much larger than that in CHO-K1 or HeLa, and their size is smaller, single-vesicle analysis of lifetimes is extremely difficult due to unreliable image segmentation. Therefore, we estimated the fluorescence lifetime for the cell as a whole, although visually, in some cases, a significant scattering of this parameter across certain intracellular regions is visible. From each image, a histogram of the distribution of fluorophore luminescence lifetimes was obtained (Figure 4b,d), with the median lifetime indicated in every image. These data were used to evaluate the effect of hypoxia, as well as the effect of BafA1 inhibitor, on the luminescence lifetimes of the sensor emitters in enMSCs. We found that the lifetime of FITC was of ca. 4.0 ns and changed very slightly for all variants, which is reflected in the histograms (Figure 4b) obtained from the representative images presented in Figure 4a, and in the box plots summarizing the expanded data obtained on the basis of the analysis of 30–50 cells in each of three independent experiments (Figure 4c). In addition, in the cells without incubation with the sensor, the background fluorescence lifetime estimated using the same imaging parameters turned out to be 3.9 ns, i.e., close to the lifetime values of endogenous fluorophores in the cell, namely to the autofluorescence of NAD(P)H/NADH and FAD. Indeed, free NAD(P)H has a relatively short luminescence lifetime of 400 ps due to self-quenching, but for protein-bound NADH it ranges between 1.0 and 4.0 ns [32,33]. FAD exists in two conformations, one of which has luminescence lifetime of the order of several ps and the other one has luminescence lifetime of about 2–3 ns, which is confirmed both in a neutral aqueous solution and in cells [34,35]. In turn, the fluorescent dye FITC in phosphate buffer has luminescence lifetime of 4.1 ns (3.0–4.0 ns depending on pH) [36]. Thus, the intrinsic background autofluorescence of enMSCs does not allow adequate estimation of the FITC fluorescence lifetime under normal conditions and upon variations in internal environment in the compartments under study.

However, for the PLIM images (Figure 4a, lower panel), distribution histograms (Figure 4d) and box plots (Figure 4e), it can be seen that the sensor shows a fairly good sensitivity to the level of oxygen in monolayer cells upon changing the oxygenation state. When the cells are grown under normal conditions (20% oxygen, 5% CO_2_), the averaged median lifetime of Ir in endolysosomes was about 3.2 μs, while under 30 min hypoxia (air was completely substituted with nitrogen while the CO_2_ level remained unchanged), it reached 5.8 μs. The large range of lifetime variations makes it possible to reliably assess changes in oxygen consumption in lysosomes, which is obviously associated with a change in the level of available oxygen in the cytoplasm. The reason for slight but reliably detectable increase in Ir emitter lifetime upon inhibition of endolysosomal acidification with BafA1 needs more detailed study.

**Figure 5:**
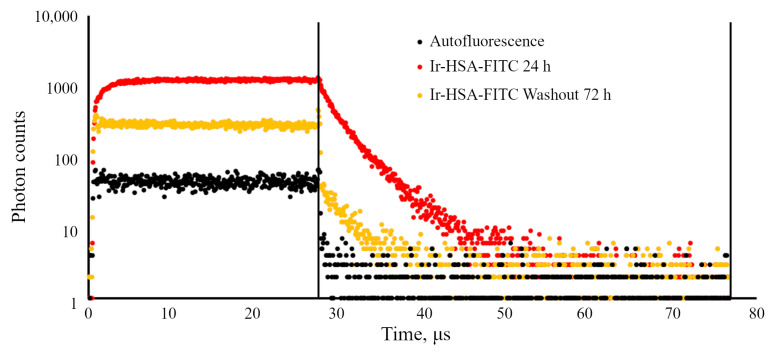
Ir decay curves obtained from PLIM images of enMSCs contained Ir-HSA-FITC (20 μm) after 24 h of accumulation and after subsequent washout for 72 h after media change. Decay curve for the cells without Ir-HSA-FITC are also presented (autofluorescence).

Importantly, when analyzing monolayer cells with Ir-HSA-FITC using PLIM, it was revealed that 72 h after washout, the sensor is still present in the cells, but at a very low level (Figure 3b), since the PLIM signal from the loaded and washed cells was still recordable in contrast to the cells without a sensor. Approximation of the time-resolved experimental data in this case occurs, as a rule, with a large uncertainty ($\chi^{2}$ up to 8) due to the low signal-to-noise ratio. An example of experimental luminescence decay curves collected from the cells after incubation with Ir-HSA-FITC for 24 h and 72 h after washout is shown in Figure 5. To enable a quantitative comparison of the phosphorescence signals, the conditions for excitation and detection of phosphorescence were kept the same in all experiments. Also, to correctly compare the amount of accumulated sensor in the cells, the phosphorescence intensity, calculated as the area under the curve of time-resolved signals, was normalized to the cell area. Analysis of the normalized signals of cell phosphorescence before and after washout showed that within 72 h, on average, the signal decreases by eight times or more that corresponds to approximately three division cycles. Thus, the low amount of the sensor in monolayer enMSCs 72 h after washout produces signal, which is insufficient for correct evaluation of lifetime values. Nevertheless, in enMSCs after 24 h incubation, the phosphorescent component of the dual sensor works adequately and provides reasonable agreement with the PLIM data reported earlier for the same sensor in CHO-K1 cells [26].

### 2.3. Detection of Ir-HSA-FITC Sensor Luminescence in Spheroids Derived from enMSCs

**Figure 6:**
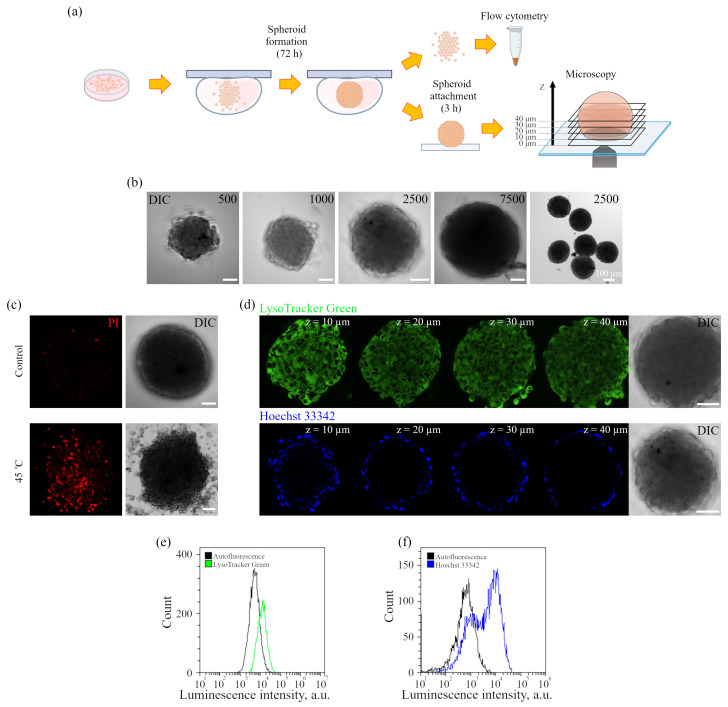
Preparation and characterization of enMSC spheroids. (**a**) Scheme of spheroid formation by the hanging drop method. The analyzed optical sections are presented in the picture on the right. (**b**) DIC images of spheroids obtained from different numbers of cells (indicated in images). Scale bars 50 μm. (**c**) Confocal microscopy of spheroids stained by PI in control (20 min, upper image), and 4 h after heat treatment as positive control for dead cells (lower image). Each image corresponds to optical cross-section at 40 μm along the z-axis. Scale bars 50 μm. (**d**) Confocal and DIC images of spheroids, stained with or Hoechst 33342 (1.6 μm, 20 min). Each image corresponds to cross-section from the bottom to the upper part at an interval of 10 μm along the z-axis. Scale bars 50 μm. (**e**,**f**) Distributions of the fluorescence intensity of the enMSC population in the control spheroids (autofluorescence) and after LysoTracker Green (**e**) or Hoechst 33342 (**f**) staining of spheroids (flow cytometry analysis).

We also analyzed the behavior of the Ir-HSA-FITC sensor in enMSC-derived spheroids as a 3D multilayer culture model. The hanging drop technique was chosen to form the spheroids. Spheroids from a drop were put on a coverglass and so «the bottom» means the layer of spheroid that contacts with it (zero level), as is shown in Figure 6a. By varying the density of cell suspension in the droplets, it is possible to control the spheroid size (Figure 6b). Moreover, it can be seen that this method allows obtaining circular spheroids of similar sizes. The studies were carried out on spheroids composed of about 2500 cells with an average diameter of 200 μm. These parameters were chosen to avoid the appearance of dead cells in the inner layers, which is typical for larger spheroids with the diameter of more than 500 μm [37,38,39]. Under these conditions (the formation of spheroids for 3 days without changing the medium), in contrast to actively proliferating monolayer enMSCs, the cells in spheroids do not divide. To evaluate cell viability in spheroids, we stained them with a vital dye, propidium iodide (PI), and compared the staining pattern with that observed after heating up to 45 °C. One can see the absence of PI-stained dead cells in the control, indicating the native state of the cells in the spheroid, while after heat shock, the dead cells are distributed across the entire spheroid (Figure 6c).

Next, the possibility of penetration of fluorescent dyes into the spheroid was investigated. It has been shown that after 20 min of incubation with intravital late endosome and lysosome dye LysoTracker Green, that penetrates the cell membrane and accumulates in acidic compartments, it evenly stains cells over the volume of the spheroid, which can be seen in the Z-series optical sections from bottom up to 40 μm deep (Figure 6d). It should be noted that, due to light scattering, luminescence in spheroids decreases with the depth and 40 μm is usually a limit for the signal recording. When the cells were incubated with the dye Hoechst 33342 for 20 min, which stains the cell nuclei in vivo, the staining was detected only in the outer layer of the spheroid (Figure 6d). These data were confirmed by flow cytometry of the cell suspension obtained from LysoTracker Green- or Hoechst 33342-stained spheroids (Figure 6e,f). Uniform staining of the spheroids with LysoTracker Green is reflected in a full shift of fluorescence intensity peak of the enMSC population in comparison with autofluorescence of the control spheroids, whereas staining with Hoechst 33342 revealed two populations of cells, where one part coincided with the control cells, while another part was represented by stained cells. The data on the Hoechst staining of spheroids are consistent with those obtained earlier [40]. The Hoechst dye diffusion method for separating inner and outer cells in a multicellular spheroid using flow cytometry was first described in 1982 [41]. Thus, fluorescent dyes can penetrate the spheroid in different ways, but it is important that the inner layers of cells in spheroids of current size can be recorded. Oxygen is a small molecule that may easily diffuse into the mass of spheroid; however, it, unlike the above substances, is actively metabolized by cells during cellular respiration, which leads to the appearance of oxygen gradient as it moves deep into the tissue.

**Figure 7:**
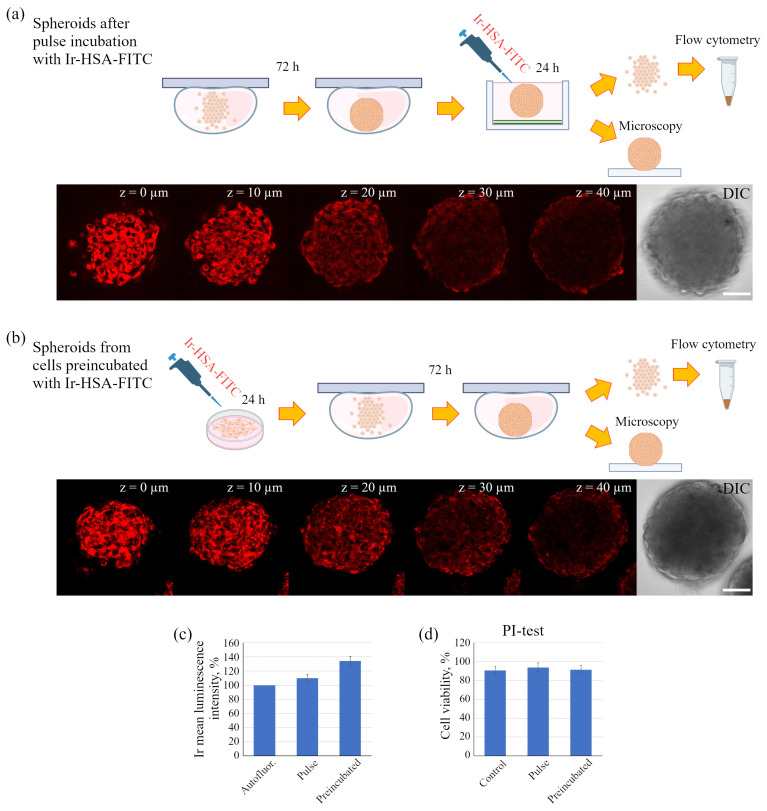
Two protocols of spheroid incubation with the sensor. (**a**) Pulse incubation of spheroids with Ir-HSA-FITC (20 μm). The cells formed spheroids for 72 h and then the sensor was added for next 24 h. (**b**) Preincubation of enMSCs with Ir-HSA-FITC (20 μm, 24 h) followed by the formation of spheroids for 72 h. Confocal and DIC images of spheroids are presented; each image corresponds to optical cross-section from the bottom to the upper part at an interval of 10 μm along the z-axis. Scale bars 50 μm. (**c**) The histograms of mean luminescence intensity per cell are presented. The value of the cell luminescence without Ir-HSA-FITC indicates their autofluorescence and is taken as 100%. The results are presented as a mean ± SEM. Three independent experiments were carried out. (**d**) Viability (PI test) of enMSCs inside spheroids before (control) and after pulse and preincubation of spheroids with Ir-HSA-FITC (in % of total cell number in probe). The results are presented as a mean ± SEM. Three independent experiments were carried out.

We performed experiments with the dual sensor in spheroids using two different protocols. In the first one, the preformed 3D spheroids were incubated with Ir-HSA-FITC (20 μm) for 24 h (pulse, Figure 7a), while in the second, 2D monolayer enMSCs were preincubated with Ir-HSA-FITC for 24 h followed by the formation of spheroids for next 72 h in sensor-free medium (preincubated, Figure 7b). At the same time, in the first case, the supply of a sensor from extracellular environment did not guarantee uniform saturation of the spheroid volume with the sensor, while in the second case, all cells in the spheroid had to contain the same quantity of the sensor initially. The analysis was carried out only in the Ir luminescence channel, since the level of background fluorescence in the FITC luminescence range was even higher in spheroids than in monolayer enMSCs and completely ruled out using of the green channel. On Z-series (optical sections was taken from bottom up to 40 μm), one can see that in both cases the Ir luminescence was recorded inside the spheroid and was localized precisely intracellularly with the absence in the intercellular space (Figure 7a,b). Flow cytometry also confirmed the presence of the sensor in the cells, but showed that the spheroids from the cells preincubated with Ir-HSA-FITC contained more Ir-HSA-FITC compared to the spheroids after pulse incubation (Figure 7c). Note that, according to PI-test, saturation of spheroids with Ir-HSA-FITC did not damage cells inside the spheroid (Figure 7d). Importantly, the monolayer enMSCs lost Ir luminescence during 72 h of incubation in the sensor-free medium, while in cells of spheroids, formed during the same 72 h from enMSCs preincubated with sensor for 24 h, Ir luminescence was perfectly detectable (compare Figure 3b and Figure 7b,c). Since there is no cell division in spheroids in our experiments, in contrast to a 2D monolayer culture, the decrease in the amount of the sensor in monolayer cells is most likely due to the dilution of sensor-bearing endolysosomes during cell divisions, rather than the release of the sensor or its hydrolyzed components into the surrounding space.

### 2.4. Characterization of Ir-HSA-FITC Sensor in enMSC Spheroids by Time-Resolved Microscopy

**Figure 8:**
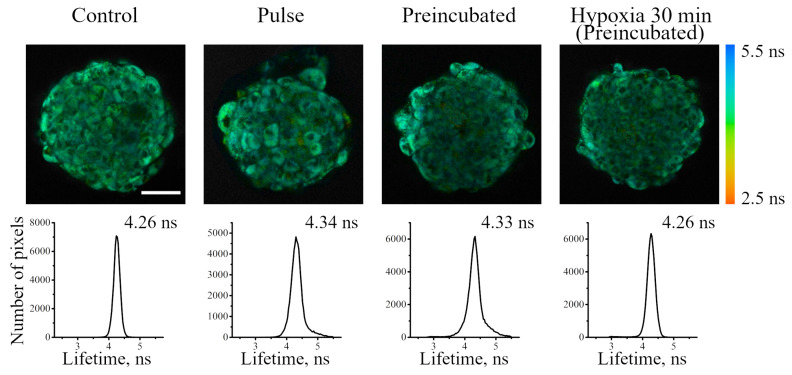
FLIM images of enMSC spheroids incubated without or with Ir-HSA-FITC (20 μm, 24 h) according to pulse or preincubated protocols. Preincubated spheroids were transferred into hypoxic medium for 30 min. Each image corresponds to optical cross-section at 30 μm along the z-axis. Scale bar 50 μm. The bottom row shows the distributions of FITC fluorescence lifetimes and median values for each case.

**Figure 9:**
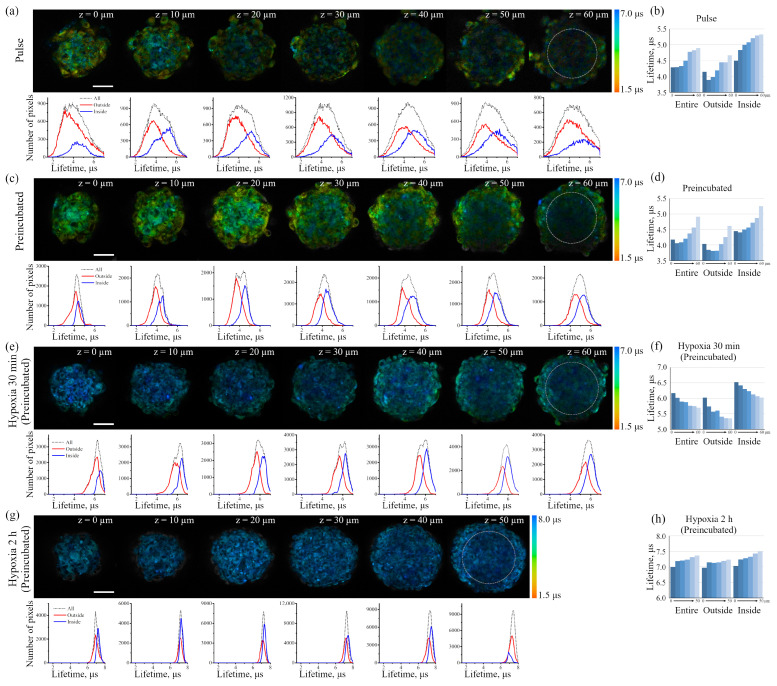
PLIM images of enMSC spheroids incubated with Ir-HSA-FITC (20 μm) according to pulse (**a**) or preincubated (**c**,**e**,**g**) protocols. Preincubated spheroids were transferred into hypoxic medium for 30 min (**e**) and 2 h (**g**). Each image corresponds to cross-section from the bottom to the upper part at an interval of 10 μm along the z-axis. Scale bars 50 μm. Under each image, the distributions of phosphorescence lifetimes for the entire spheroid and outside/inside regions are presented. The principle of outside/inside area selections is presented at the 60 μm image section. (**b**,**d**,**f**,**h**) Values of the median distributions of Ir phosphorescence lifetimes for the entire spheroids and their outside/inside regions corresponding to images from (**a**,**c**,**e**,**g**).

The sensor in spheroids was also characterized using the combined FLIM/PLIM approach. As in the case of monolayer cells, the FITC fluorescence lifetime was quite similar to that of intrinsic fluorescence, and was about 4.3 ns (Figure 8). This observation prevents any conclusions concerning the level of pH in the studied systems. Therefore, we investigated the effect of culture conditions by using only Ir phosphorescence lifetime (Figure 9). Figure 9a,c shows PLIM images of spheroids from Z-series optical sections from the bottom up (outer cell layer) to 60 μm (the layer just lower the middle of a spheroid) after incubation with Ir-HSA-FITC. Below each image, a histogram of the distribution of Ir luminescence lifetimes from the entire diameter of the spheroid (gray curve) is presented. Similar results were obtained for the both used protocols.

It was shown that as we move from the bottom of the spheroid to its central part, the value of the median of Ir phosphorescence lifetime in each optical section increases from 4.30 to 4.90 μs for the spheroids obtained after pulse incubation with Ir-HSA-FITC (Figure 9b) and from 4.06 to 4.91 μs for the spheroids obtained from preincubated enMSCs (Figure 9d). Since oxygen quenches the sensor phosphorescence, the lifetime gradient indicates a decrease in the amount of oxygen in the cells of the spheroid upon movement to its center.

We considered it reasonable to analyze phosphorescence lifetime (i) in the outer shell, i.e., cells close to the surface of the spheroid (outside); and (ii) in the central area of the spheroid (inside); and determined the lifetimes for each region separately. We identified the inner zone, represented by a circle in the images of 60 μm sections. It should be taken into account that the shape of the spheroids differs from the ideal sphere, and the position of the cells in the outer layer also differs. As a result, such a division turns out to be rather conditional, but allows a rough estimate of the cell parameters at different levels of the spheroid. In this case, the histograms show a clear difference in the distributions of Ir phosphorescence lifetimes in the two regions (Figure 9a,c; red and blue curves), with the median lifetimes in the outer shell 15% shorter than in the central area (Figure 9b,d), which also reflects the difference in the level of oxygen availability and/or its consumption. It should also be noted that the peaks in the distributions of phosphorescence lifetimes in the cells preincubated with the sensor are much narrower than in pulse-stained ones that indicates a greater homogeneity of the sensor response when using the preincubation protocol. Figure 9e shows spheroids from the cells preincubated with Ir-HSA-FITC, which were analyzed after 30 min of hypoxia, when the oxygen level in the incubation medium had just dropped to a minimum. Both the images and the histograms of lifetime distributions show a significant increase in the median Ir phosphorescence lifetime up to 6.5 μs (Figure 9e,f). The observed gradient of variations in the median lifetime across slices is opposite to that observed in normoxia, which is explained by the fact that the outer layer loses oxygen faster, and the sensor responds to this. At the same time, the central area of the spheroid has not yet been completely exposed to hypoxia by 30 min incubation in oxygen-free medium. With longer exposure to hypoxia (2 h), the phosphorescence lifetimes display homogeneous distribution for all cells of the spheroid and reached the value of ca. 7 μs (Figure 9g,h).

Comparing the data on phosphorescence lifetime gradients in spheroids under normoxia and hypoxia, it is worth noting that, under normal conditions, the inner layers of cells exist under conditions of incomplete hypoxia which, nevertheless, allows them to function for quite a long time. It is obvious that such situation should also be typical for tissue cells in deep layers under physiological conditions.

## 3. Discussion

MSCs are currently considered promising candidates for cell therapy and regenerative medicine, and one of the main areas of research is the development of tools that allow monitoring of such key parameters of cellular homeostasis as oxygen consumption and lysosomal acidification. These two processes may be interrelated, since MSC senescence is accompanied by a decrease in lysosome acidification which, in turn, leads to an increase in reactive oxygen species production and a number of other consequences [14]. Dysfunction in both acidification and oxygen consumption is also observed in neoplastic processes and neurodegenerative diseases [10,42].

It was established that MSCs function in tissues under conditions of hypoxia, but the exact hypoxia level can determine cell survival or death. The control of the oxygen level and its changes, as well as the level of acidification of lysosomes in tissue cells, is currently an extremely urgent task, which has stimulated numerous studies devoted to the development and verification of various biosensors aimed at the measurement of oxygen concentration and pH in biological systems. Phosphorescence of complexes based on iridium, ruthenium and platinum, which is quenched by oxygen, makes these compounds excellent oxygen sensors [20,43,44]. Various compounds based on fluorescein, cyanine and other fluorophores, as well as nanoparticles, are used as pH sensors [45,46].

In this work, we used a dual pH/O_2_ sensor, Ir-HSA-FITC, in which the pH-sensitive FITC is combined with the O_2_-sensitive iridium complex through conjugation of both chromophores to HSA [26]. The presence of a large protein component determines the uptake of this complex only by endocytosis and, in principle, makes it possible to simultaneously determine the level of pH and oxygen in vesicles of the endolysosomal system of the cell which, in itself, is an interesting scientific problem. Although there is an evidence of a clathrin-dependent receptor-mediated pathway for albumin internalization [47], the dynamics of sensor accumulation in cells, determined using the flow cytometry method (Figure 3), suggests that the sensor behaves like a fluid-phase marker, which is characterized by slow entry through all endocytic portals followed by accumulation in late endosomes and lysosomes. Indeed, it should be taken into account that the internalization of albumin through binding to proteins of the scavenger receptors family is typically restricted to the cells of the immune system [28,31].

The data obtained, however, show that in monolayer enMSCs, the FITC signal is not clearly detected by confocal microscopy and makes single vesicle analysis impossible in this type of cell. In our opinion, there may be several reasons for this observation: firstly, enMSCs have a very high level of autofluorescence in the range of FITC signal emission, and secondly, the endolysosomal apparatus of enMSCs is represented by a very large number of small vesicles distributed evenly throughout the cytoplasm of highly spread out cells. This indicates that, in particular, in enMSCs fusions of endosomes lead to an insignificant concentration of cargo, in comparison with the cells of HeLa or A549 type in which a strong concentration of cargo occurs in the early stages of endocytosis. The sensor internalized by enMSCs enters endosomes through all endocytic portals, but the low concentration in early endosomes with a pH of 6.8–6.0 makes it impossible to detect the signal by confocal microscopy. Within 24 h, when the sensor reaches lysosomes and concentrates there, the pH level typical for these compartments (5.5–4.5) leads to partial quenching of FITC fluorescence that was confirmed by the data (Figure 1b) on the co-localization of sensor chromophores (M1 = 0.65 ± 0.03 and M2 = 0.48 ± 0.02). It is interesting to note that in the presence of BafA1, which abolishes acidification of endosomes and thus leads to the increase in the fluorescence intensity of quenched FITC molecules, a much larger number of bright vesicles with the corresponding FITC signals was detected. However, even in this case, the co-localization of FITC and Ir signals is incomplete with Manders’ coefficients comparable to the above-mentioned values (compare Figure 1b and Figure 2a). We assume that this effect is associated with the degradation of HSA in lysosomes that could result in separation of the FITC and Ir chromophores to give independent localization of the pH and oxygen sensors. Since lysosomes are able to exchange contents during multiple fusions and fissions of endolysosomes, the FITC:Ir ratio, which was initially close to 1:1, can be significantly violated.

However, both chromophores remain in lysosomes. Firstly, the most expected cleavage products containing emitters are their conjugates with short peptides or single amino acids, mainly lysine, since this amino acid is the most abundant source of primary amino groups, the target conjugation functions for both FITC and Ir complex [48]. We suppose that the complete detachment of emitters from peptides or amino acids is unlikely due to their much lower susceptibility to cleavage by various lysosomal hydrolytic enzymes. Linking of the emitters to one or several amino acid residues makes them rather hydrophilic and, therefore, can suppress their lysosomal escape. Second, FITC is known as hydrophilic and thus as a membrane non-permeable compound. As a result, we have found rather high co-localization of both emitters with LysoTracker Deep Red (LTDR), with Manders’ co-localization coefficients (M1) of 0.84 ± 0.02 and 0.79 ± 0.02 for FITC and Ir complex, correspondingly (Figure 1b), even at 24 h incubation. Additionally, we have not observed the emission of Ir complex inside the cytoplasm, which also corroborates our conclusion regarding the primary localization of both emitters inside lysosomes.

As was stated above, it was not possible to use Ir-HSA-FITC as a FLIM pH sensor in enMSCs due to high autofluorescence typical for these cells (Figure 4a and Figure 8). Nevertheless, this conjugate contains two independent emitters and, thus, it could be used as a ratiometric probe like was recently demonstrated for another pH-sensor based on albumin [49]. Unfortunately, this possibility also seems unreliable because of two main reasons mentioned above: (i) the Ir-HSA-FITC probe can be (at least partially) cleaved in lysosomes, and (ii) the Ir:FITC ratio can be significantly deviate from the starting 1:1 value due to numerous fusions and fissions of endolysosomal vesicles. To avoid this drawback in future, further research should be aimed at the development of constructions stable towards lysosomal degradation.

It is important to stress that flow cytometry approach seems to be more sensitive than confocal microscopy, but can result in misinterpretations due to the fact that monolayer cells in this case must be transferred into suspension before analysis, which can lead to rapid changes in both endolysosomal acidification and, what is more realistic, in the redox status of cells. We have found here that Ir chromophore lifetime reacts rather fast onto the changes in oxygen availability, even when the emitter is localized in lysosomes. Nevertheless, this approach seems to be the most adequate for determining the dynamics of changes in the amount of internalized sensors per cell by fluorescence intensity estimations.

On the other hand, there could be another way to quantitatively detect the FITC signal in individual endosomes by using as a carrier the protein compounds, which are able to enter the cells itself or through interaction with some intermediate via highly efficient receptor-mediated endocytosis. Streptavidin, capable of binding biotinylated ligands or antibodies to efficiently internalized cytokine receptors, and similar high affinity systems, can be imagined as a more promising alternative for HSA. Such carriers are rapidly concentrated in endosomes as early as 5–15 min after internalization due to active homotypic fusions [50,51], while the pH in such endosomes does not yet fall below 6.0, which would give a stronger FITC emission and higher signal-to-noise ratio.

The iridium component of the sensor has demonstrated good sensitivity to hypoxia in both cell monolayers and spheroids loaded with the sensor according to both protocols (see above). In contrast to FITC, the Ir luminescence intensity, which is not very strong by itself, remains easily detectable and strongly different from background fluorescence, giving substantial variations in its lifetime (from 3–4 μs to 6–7 μs) upon changes from normoxia to hypoxia, respectively. Such a wide range of changes makes it possible to detect the effects of hypoxia with a high degree of reliability. However, given the peculiarities of the endolysosomal apparatus of enMSCs, segmentation of PLIM images with a large number of small vesicles is a significant obstacle for single vesicle analysis, so we averaged the data per cell. The obtained spheroids contain about 2500 cells each, and display an average diameter of about 200 μm (Figure 6), which made it possible to avoid cell death in the internal space, which is believed to be characteristic of spheroids with a diameter of more than 500 μm [37,38,39]. At the same time, such spheroids demonstrate cell heterogeneity and metabolic gradients of oxygen, glucose and ATP, which appear in spheroids only when they are larger than 150 μm. The obtained data show that the spheroids do not have a necrotic nucleus and all the cells in them are alive (Figure 6c and Figure 7d), and substances such as PI or LysoTracker Green freely penetrate the inner layers and are fairly evenly distributed among all cells. Since the sensor has a rather large size due to albumin, one would expect difficulties in its penetration into the inner layers of spheroid cells in the pulse staining protocol. A comparison of the distribution of the Ir signal in spheroids, all cells of which were pre-loaded by the Ir-HSA-FITC and hence contained the same amount of the sensor, with those that were incubated with the sensor after their formation, showed that the sensor penetrates the cells of all layers; however, in the second case, the PLIM signal was more heterogeneous, with wide lifetime distribution peaks. Nevertheless, the median values coincided with those in the preincubated cells (see the histograms under the images of the corresponding cells in Figure 9a,c). It is important to take into account that the pulse staining approach is much more compatible with various practical applications, e.g., for the analysis of the oxygen level in tissues, where it could give reliable results.

However, for in vitro experiments, it is preferable to use a protocol with preincubation. It should also be emphasized that the fluorescent signal of cells in the inner layers of the spheroid is practically not detected at depths of more than 40 μm due to light absorption/scattering, while the PLIM signal is reliably recorded up to 60 μm due to higher sensitivity of the corresponding detectors. It also should be taken into account that, in contrast to luminescence intensity, PLIM data are more reliable due to lifetime independence of the sensor concentration. The advantage of phosphorescence lifetime visualization was directly demonstrated in a detailed comparison of the two approaches performed on spheroids from the Colon26 mouse colon carcinoma cell line using a platinum-based O_2_ sensor [52].

Data on the accessibility of the inner layers of the spheroid to small molecules support the idea that a small molecule such as oxygen should also be distributed evenly in the depth of the spheroids; however, O_2_ is not simply accumulated in the cells, but is involved in the metabolism, which results in its consumption. Indeed, PLIM analysis has demonstrated lifetime gradient (from about 4.5 to 5.3 μs) in spheroids, indicating a decrease in oxygen availability in the inner layers (Figure 9a–d). This phenomenon can be interpreted as the appearance of a stationary state that emerges as the result of interplay of two processes: (i) O_2_ diffusion into the spheroid, and (ii) irreversible oxygen consumption inside cells. This interplay leads to lower oxygen accessibility and, hence, its lower stationary concentration, in inner parts of spheroids. The gradients of sensor lifetimes in enMSC spheroids were similar to those obtained in spheroids of other origin using both Ir- and Pt-based oxygen sensors [53,54,55]. To understand how quickly the sensor in cell lysosomes reacts to hypoxia, we replaced the air with nitrogen in a special chamber, and kept the spheroid under such conditions for 30 min before PLIM recording (Figure 9e,f). In this experiment, we observed an “inverted” lifetime gradient, indicating moderate increase in lifetimes in the outer layer of cells (ranging from 6.0 to 5.3 μs), and lifetime increase to higher values (ranging from 6.5 to 6.0 μs) in the inner layers of the spheroids.

Comparing normoxia with such a short-term (30 min) hypoxia, it must be stressed that the cells in the inner layers maintain “mild hypoxia”, which is characterized not by the complete inaccessibility of oxygen, but only by its reduced level. Keeping the cells under conditions of complete hypoxia for 2 h showed that, eventually, all the cells of the spheroids lost oxygen, and this conditional level of “absolute hypoxia” corresponds to the sensor phosphorescence lifetime of about 7.5 μs measured throughout the entire spheroid (Figure 9g,h). This value is in good agreement with the Ir lifetimes measured for the same sensor in deeply deoxygenated phosphate-buffered saline at pH 7.4 [26].

The obtained results indicate that the sensor can be successfully used to assess the dynamics of changes in the oxygen level in cell lysosomes under different external stimuli, and it, in turn, reflects the oxygen availability in the cytoplasm.

It is known that cells in tissues are subject to changes in oxygen concentration and, consequently, in the level of hypoxia. For example, cyclic fluctuations in oxygen concentration have been demonstrated in tumors in vivo due to changes in erythrocyte flow, as well as in vascular remodeling, and thermoregulation [56]. It is very important to have experimental models that (i) adequately mimic in vivo tissue oxygen fluctuations and (ii) allow simultaneous control of O_2_ levels. The spheroids loaded with PLIM oxygen sensors appear to be an experimental model that bridges the gap between standard experimental protocols and clinical settings.

## 4. Materials and Methods

### 4.1. Cell Culture

Human endometrial mesenchymal stem/stromal cells (enMSCs) were isolated from a desquamated endometrium of menstrual blood from healthy donors [57] and demonstrated properties typical for the mesenchymal stem cell cultures. enMSCs were cultured in DMEM/F12 medium (GIBCO, Waltham, MA, USA) supplemented with 10% fetal bovine serum (GIBCO, Waltham, MA, USA), 1% L-glutamine, and 1% penicillin-streptomycin (GIBCO, Waltham, MA, USA), in an atmosphere of 5% CO_2_ at 37 °C. The cells from the third to ninth passages were reseeded twice a week at the split ratio 1:3. These cells are characterized by high rate of cell proliferation (doubling time 22–23 h).

For experiments, the cells were harvested by trypsinization and plated on Petri dishes with glass coverslips (Nunc, Thermo Scientific, Rochester, NY, USA) or on 35 mm imaging μ-dishes with a polymer coverslip bottom (Ibidi GmbH, Gräfelfing, Germany) or 12-well plates (Nunc, Thermo Scientific, Rochester, NY, USA) in DMEM/F12 medium, supplemented with 10% fetal bovine serum, or were used to form spheroids. The experiments were performed at 60–70% confluence 48 h after seeding.

The experiments were approved by the Ethics Committee of the Almazov National Medical Research Centre (Saint-Petersburg, Russia) and performed in accordance with the institutional guidelines. All cell donors signed an informed consent for voluntary participation.

### 4.2. Spheroid Formation

Spheroids (3D-cultures) were formed from monolayer enMSCs (2D cultures) using the hanging drop technique. A total of 2500 cells in suspension per 35 μL of DMEM/F-12 medium containing 10% fetal bovine serum were placed in drops on the cover of 5 cm Petri dishes (Nunc, Thermo Scientific, Rochester, NY, USA) and inverted. During the next 72 h, cells in hanging drops spontaneously formed spheroids.

For confocal microscopy, already formed spheroids were placed in dishes with a polymer coverslip bottom with full growth medium and were left for 3–4 h to ensure attachment and immobility of spheroids.

For the flow cytometry assay, the single-cell suspension from spheroids was obtained. For this purpose, spheroids with culture medium were transferred to a 15 mL conical tube and centrifuged for 3 min at 1500 rpm. After centrifugation, the supernatant was removed; 10 mL of phosphate-buffered saline solution was added, and the cells were centrifuged again. Then, the supernatant was removed, and spheroids were incubated with 0.05% trypsin/EDTA solution for 5 min at 37 °C. After the incubation was completed, the cell suspension was gently pipetted 2–3 times to achieve spheroid dissociation. Finally, a three-fold volume of the culture media was added for trypsin inactivation.

### 4.3. Incubation with Ir-HSA-FITC Sensor

The Ir-HSA-FITC sensor based on human serum albumin (HSA) conjugated with a pH-sensitive fluorophore (FITC) and an O_2_-sensitive phosphor represented by cyclometalated iridium complex ${\left[\mathrm{Ir}{\left(\mathrm{NC}\right)}_{2}\left(\mathrm{NN}\right)\right]}^{+}$ used in this study were synthesized and characterized according to the methods described in our previous publication [26].

Ir-HSA-FITC was dissolved in the water at concentration of 250 μm and added to the enMSC monolayers in a final concentration of 20 μm. After incubation with the probe for indicated time, cells were washed with fresh media and analyzed.

The sensor was introduced into spheroids according two protocols: in the first one, already formed spheroids were transferred to dishes coated with a nonadhesive substrate (2-hydroxyethyl methacrylate, Sigma-Aldrich, St. Louis, MO, USA) and cultured in full growth medium with Ir-HSA-FITC for the next 24 h (pulse); in the second one, 2D monolayer enMSCs were preincubated with Ir-HSA-FITC for 24 h followed by the formation of spheroids in the absence of the sensor (preincubated).

### 4.4. Intracellular Compartment Identification

For vital staining of lysosomes and late endosomes in enMSCs or spheroids, LysoTracker Deep Red or LysoTracker Green DND-26 (Invitrogen, Eugene, OR, USA) at a concentration of 50 nM was used. LysoTracker was added into the culture medium for 20 min prior to confocal imaging. For vital staining of nuclei in spheroids, Hoechst 33342 (Invitrogen, Eugene, OR, USA) was used at a concentration of 1.6 μm for 20 min before analysis.

### 4.5. Cell Treatments

Bafilomycin A1 (BafA1, Sigma-Aldrich, St. Louis, MO, USA), a specific inhibitor of vacuolar type H^+^-ATPase, at a concentration of 100 nM was added for 30 min to the cells, preliminarily incubated with Ir-HSA-FITC (20 μm) for 24 h.

Experiments were carried out under a normal oxygenated atmosphere, containing 5% CO_2_, 37 °C (normoxia). In case of hypoxia, air was completely substituted with nitrogen while the CO_2_ level remained unchanged; the cells or spheroids were incubated under such conditions for 20–30 min and for 2 h.

### 4.6. Evaluation of Cell Metabolic Activity by MTT Test

enMSCs were seeded in 96-well plates at 10^4^ cells per well and allowed to grow for 24 h. After that, Ir-HSA-FITC were added to cells for 24 h at concentrations of 0–80 μm. At the end of the incubation, Ir-HSA-FITC was washed out, the medium was exchanged for fresh DMEM/F12 without phenol red (100 μL/well), and then, 10 μL/well of 3-(4,5-dimethylthiazol-2-yl)-2,5-diphenyltetrazolium bromide reagent (MTT, 5 mg/mL, Invitrogen, Eugene, OR, USA) was added. After 4 h of additional incubation, the media were removed, and 50 μL/well of DMSO was added to solve the formed formazan crystals produced by the metabolic active cells, and a Thermo Scientific Multiskan FC microplate format photometer (Thermo Fisher Scientific, Waltham, MA, USA) determining the optical absorption at a wavelength of 570 nm was used to measure the cell viability.

### 4.7. Analysis of Cell-Associated Fluorescence by Flow Cytometry

Flow cytometry analysis was carried out by CytoFLEX cytometer (Beckman Coulter, Brea, CA, USA) at a maximum sample feed rate (1 μL/s) for 100 s. The data obtained were analyzed using the CytExpert 2.0.0.152 software (Beckman Coulter, Brea, CA, USA) and are presented as diagrams and histograms.

Laser excitation wavelengths at 405 (for Ir) and 488 (for FITC) nm were used to estimate the amount of Ir-HSA-FITC associated with cells in monolayer culture and in spheroids. Luminescence was recorded using combination of 610/20 and 660/10 BP filters for Ir and 525/40 BP filter for FITC.

Hoechst 33342 was excited at 405 nm and registered with 450/45 BP filter; LysoTracker Green was excited at 488 nm and registered with 525/40 BP filter; PI was excited at 488 nm and registered with 690/50 BP filter. Autofluorescence of cells was analyzed under the same conditions for each case.

### 4.8. Estimation of Cell Survival with PI Test

Living cells have membranes that are still intact and exclude propidium iodide (PI, Sigma, St. Louis, MO, USA) that easily penetrates the damaged, permeable membranes of non-viable cells. PI at a concentration of 50 μg/ml was added for 1–2 min to the suspensions of control cells and the cell suspension obtained after disintegration of spheroids incubated with Ir-HSA-FITC. Then, the fluorescence of cells was analyzed by flow cytometry. The results were expressed in the proportion of live cells (not stained with PI) relative to the total number of cells in each sample.

Spheroids were incubated with PI (40 μg/mL, 20 min at 37 °C) and analyzed using a confocal microscope. In this case, a positive control visualization of the dead cells in spheroids was achieved using PI staining performed 4 h after the stress insult by heat shock (spheroids were incubated at 45 °C for 30 min in the water bath).

### 4.9. Confocal Microscopy

Cell imaging was performed using an Olympus FV3000 laser scanning confocal microscope (Olympus, Tokyo, Japan). The samples were observed with a 40/1.42× oil immersion objective, obtaining images of 1024×1024 pixels. Images were captured in one, two or three spectral channels in sequential scan mode, with only one laser operating at a time to avoid spectral overlap, and in a channel of differential interference contrast in transmitted light (DIC). Z-series optical sections for monolayer cells were taken at 0.5 μm steps from bottom to top (14–16 sections) and for spheroids at 10 μm steps from bottom to top (5–7 sections). When indicated, the luminescence of Ir-HSA-FITC was studied in spectral scanning mode (Mode/xyλ) in the range of 505–675 nm with a step of 5 nm.

The dual emission of Ir-HSA-FITC was excited separately with 405 and 488 nm lasers. The emission of iridium chromophore was recorded in the 570–670 nm range and FITC fluorescence signal was recorded in the 500–550 nm range. In the spectral scanning mode, the emission of Ir-HSA-FITC was excited simultaneously with 405 and 488 nm lasers.

LysoTracker Green was excited at 488 nm and recorded in the 500–550 nm range. LysoTracker Deep Red fluorescence was excited at 640 nm and recorded at 650–750 nm. Hoechst 33342 fluorescence was excited at 405 nm and recorded in the range of 430–480 nm. PI fluorescence was excited at 488 nm and recorded in the range of 600–680 nm.

In each experiment, 5–10 fields containing 20–60 cells totally were imaged for each experimental point. Images were processed and analyzed using Fiji software 1.52v (National Institutes of Health, Bethesda, MD, USA). The most representative single sections from a Z-series of typical cells were selected for demonstration. For quantitative analysis, raw images were used.

### 4.10. Combined FLIM/PLIM Experiments

Combined fluorescence and phosphorescence lifetime imaging microscopy (FLIM/PLIM) of enMSCs or spheroids was carried out using a time-correlated single photon counting (TCSPC) DCS-120 module (Becker & Hickl GmbH, Berlin, Germany) integrated into the Nikon Eclipse Ti2 (Nikon Corporation, Tokyo, Japan) confocal instrument. All measurements were performed in humidified Stage Top Incubator Tokai HIT (Tokyo, Japan) at 37 °C and 5% CO_2_. Required CO_2_, air and N_2_ percentage was maintained by Tokai HIT Digital gas mixer GM-8000.

Emission was excited with a picosecond laser at 405 nm, light from the sample was divided on a beam splitter, and fluorescence and phosphorescence were recorded in two channels. FLIM was recorded in the channel with 435 nm long pass filter and 535/30 nm band pass filter and pinhole of 0.25–0.5. PLIM was recorded using 575 nm long pass filter and 630/75 nm band pass filter and pinhole of 0.25–0.5. The following settings were used: frame time 21.74 s, pixel dwell time 81.60 μs, total acquisition time 173.9 s, and image size 512 × 512 pixels. Water immersion 40× objective with zoom 5.33 for cells and 1.43 for spheroids. Fluorescence and phosphorescence lifetime distributions were calculated using SPCImage 8.1 software (Becker & Hickl GmbH, Berlin, Germany). Fluorescence decays were fitted monoexponentially with an average goodness of the fit 0.9 ≤ $\chi^{2}$ ≤ 1.3. The phosphorescence decay curves were fitted in monoexponential decay mode with an average goodness of the fit 0.8 ≤ $\chi^{2}$ ≤ 1.2. The average number of photons per curve were not less than 5000 at binning 4 (monolayer cells) or 7 (spheroids). The data are presented as distribution of fluorescence or phosphorescence lifetime collected from the scanning field, as indicated in figure legends.

### 4.11. Statistical and Co-Localization Analyses

The images were processed and analyzed using Fiji software 1.52v (National Institutes of Health, Bethesda, MY, USA). The quantitative co-localization analysis was performed using ImageJ JACoP Plugin to determine Manders’ co-localization coefficients (M), which are defined using thresholds as the sum of intensities of co-localized pixels from one channel divided by their integrated density. Thresholds were set by a visually estimated value for each channel. The results are represented as mean ± standard error of the mean (SEM).

Statistical data processing was performed using Microsoft Office Excel 2021 (Microsoft Corporation, Albuquerque, NM, USA). The graphs were built using the Origin 8.5 software (OriginLab, Northampton, MA, USA), the bar charts (mean ± SEM) and box plots using Microsoft Office Excel 2021. All results were obtained from at least three independent experiments.

## 5. Conclusions

In this work, we have evaluated the applicability of the Ir-HSA-FITC conjugate [26] for dual pH/O_2_ sensing inside endolysosomes of enMSCs grown in monolayer and in spheroids. The sensor demonstrated several strengths: it is internalized by the cells and evenly distributes across the 3D spheroids while its phosphorescent signal can be reliably recorded up to a depth of 60 μm compared to practically achievable limit of 40 μm typical for fluorescence; the iridium component of the sensor demonstrated high sensitivity to hypoxia in both cell monolayers and spheroids loaded with the sensor. However, a practical dual performance of the Ir-HSA-FITC sensor was not achieved due to strong interference of enMSC autofluorescence with the FITC signal. Another drawback relates to the sensitivity of the probe to lysosomal HSA cleavage, which could potentially compromise the assay. This possibility seems highly plausible for the two main reasons mentioned above: because the Ir-HSA-FITC probe may be (at least partially) cleaved in lysosomes and consequently, the Ir:FITC ratio may deviate from the initial value of 1:1 due to numerous fusions and divisions of endolysosomal vesicles. To avoid this drawback in future, further research should be aimed at the development of “dual sensor” platforms stable towards lysosomal degradation and to substituting of FITC by other pH-sensitive fluorophores emitting at longer wavelengths or longer luminescence lifetime. Nevertheless, the data obtained emphasize that the described problems in recording the FITC signal are characteristic of cells with a high level of autofluorescence and peculiarities of organization of the endolysosomal apparatus. Previously, we have shown that such problems are minimal in cells with low levels of autofluorescence and the ability to reliably identify individual endosomes [26]. Degradation of the sensor in lysosomes also introduces only a small interference, since the FLIM/PLIM data do not depend on the sensor concentration. Our work represents one of the first detailed analyses which demonstrated that internalizable sensor effectively enters the cells in enMSC spheroids, reliably reflects the gradient of oxygen inside the spheroids and shows the fast reaction to external hypoxia. In this respect, the sensor demonstrates potential suitability for monitoring the $O_{2}$ level variations.

Overall, the presented study provides a deep insight into various aspects of dual sensing in sophisticated cell models and gives the rationale for further improvement of dual lifetime sensors.

## Data Availability

The data presented in this study are available from the authors upon request.

## Abbreviations

The following abbreviations are used in this manuscript:

BafA1	bafilomycin A1
DIC	Differential Interference Contrast
DMEM	Dulbecco’s Modified Eagle Medium
EDTA	Ethylenediaminetetraacetic acid
enMSCs	endometrial MSCs
FITC	fluorescein isothiocyanate
FLIM	fluorescence lifetime imaging
HSA	human serum albumin
LTDR	LysoTracker Deep Red
MSCs	mesenchymal stem/stromal cells
MTT	3-(4,5-dimethylthiazol-2-yl)-2,5-diphenyl-2H-tetrazolium bromide
PLIM	phosphorescence lifetime imaging
